# Sulphite oxidase (SO) – a mitochondrial autoantigen as target for humoral and cellular immune reactions in primary sclerosing cholangitis

**DOI:** 10.1186/s12876-018-0787-x

**Published:** 2018-05-02

**Authors:** Beate E. Preuß, Christoph P. Berg, Christoph Werner, Sandra Plankenhorn, Nisar P. Malek, Reinhild Klein

**Affiliations:** 1 Department of Internal Medicine II, University of Tuebingen, Otfried-Mueller Str. 11, 72076 Tuebingen, Germany; 2 Department of Internal Medicine I, University of Tuebingen, Tuebingen, Germany

**Keywords:** Primary sclerosing cholangitis, Sulphite oxidase, Autoantibodies, Epitope mapping, Cellular immune reactivity

## Abstract

**Background:**

In a recent study we had evidence that sulphite oxidase (SO) may be a relevant autoantigen in primary sclerosing cholangitis (PSC). Aim of the present study was, therefore, to analyse humoral and cellular immune-reactivity towards SO in these patients in more detail.

**Methods:**

Sera from 53 patients with PSC (30 untreated and 23 treated with ursodeoxycholic acid [UDCA] at time of analysis), from 422 patients with different hepatic and non-hepatic disorders, and from 50 healthy individuals were tested by ELISA for antibodies against full-length-SO (SO-fl) and its three major domains expressed in *E.coli* (SO-I, SO-II, SO-III). For epitope-mapping, 29 overlapping peptides were used. Peripheral blood mononuclear cells (PBMC) were obtained from 33 PSC-patients and analysed for SO-induced proliferation, production of cytokines, and expression of the activation marker cluster of differentiation (CD) 69.

**Results:**

43% of the 30 untreated and 26% of the 23 treated PSC-patients had IgG anti-SO-antibodies predominantly reacting with SO-fl, SO-I and SO-II. Antibody-reactivity decreased after UDCA-treatment. Prevalence and reactivity of anti-SO-antibodies were significantly higher in PSC than in patients with other hepatic and non-hepatic disorders. Epitope mapping revealed no distinct immuno-dominant regions within SO. Incubation of PBMC from PSC-patients (but not from controls) with SO-antigens revealed an activation of B-cells and a T-helper cell type-2 reaction pattern (production of interleukin [IL]-13, IL-10).

**Conclusions:**

PSC-patients show humoral and cellular immune response towards SO. Antibodies may be predominantly directed against conformational epitopes. SO enhances in vitro especially T-helper cell type-2 immune-reactions, which may be pro-fibrotic. SO is a detoxifying enzyme present also in bacteria; further studies analysing its role in the aetiology and pathogenesis in PSC may, therefore, be important.

**Electronic supplementary material:**

The online version of this article (10.1186/s12876-018-0787-x) contains supplementary material, which is available to authorized users.

## Background

Primary sclerosing cholangitis (PSC) is a chronic cholestatic liver disease characterised by diffuse inflammation, fibrosis and sclerosis of intra- and/or extrahepatic bile ducts [1]. Its aetiology and pathogenesis remains still poorly understood [2–4]. In 30-70% of PSC patients antibodies to neutrophils (p- or xANCA) can be detected [5, 6]. Their target autoantigen has been elusive; cathepsin G, elastase, lactoferrin, tubulin beta isoform 5 or the bacterial protein FtsZ have been discussed [7]. The clinical relevance of pANCA as diagnostic marker is limited because they are also detectable in patients with other disorders [8, 9]. Furthermore, there is no correlation between ANCA and PSC activity [10].

Previously we showed that IgG-antibodies against the mitochondrial enzyme sulphite oxidase (SO) have a high prevalence in PSC [11]. Primarily we had reported that antimitochondrial antibodies of the subtype M4 in primary biliary cholangitis (PBC) reacted with an antigen present in a chicken liver-derived SO-fraction [12]. However, expressing SO in *E.coli* we could exclude that M4 corresponds to SO; but testing sera from patients with a variety of liver disorders against this recombinant SO we accidentally found the strong association of anti-SO with PSC [11].

SO is a ubiquitous enzyme located in the intermembrane space of mitochondria. It is a homodimer consisting of three domains (see Additional file 1): an N-terminal cytochrome b5-like heme/steroid binding, an oxidoreductase molybdopterin cofactor binding, and a C-terminal immunoglobulin-like dimerization domain. SO is involved in the conversion of sulphite to sulphate hereby detoxifying excess sulphite [13, 14]. Human SO deficiency is a fatal genetic disorder that leads to mental retardation and early death [15]. Autoimmune processes directed against SO have not yet been described in the literature.

Aim of the present study was, therefore, to see whether humoral and cellular immune reactions towards SO and its domains or distinct epitopes exist in PSC patients.

## Methods

### Patients

Fifty-three patients with PSC (21 females, 32 males; mean age 35 years, range 18-78 years) were analysed. Diagnosis was based on typical clinical and laboratory features and bile duct strictures in the endoscopic retrograde cholangio-pancreaticoscopy (ERCP). Twenty-two (42%) had pANCA in the immunofluorescence test (IFT). Detailed clinical and laboratory parameters of these patients are given in Additional file 2.

Twenty-eight of the 53 PSC-patients additionally suffered from inflammatory bowel disease (IBD), four patients developed autoimmune hepatitis (AIH) in the course of the disease, and five patients had other autoimmune diseases (Additional file 2). From 30 of the 53 patients sera were available before any therapy, 23 patients were already under therapy with ursodeoxycholic acid (UDCA) for at least six months at time of first examination. From a previous study we had evidence that UDCA-therapy decreases anti-SO-reactivity [11]. We, therefore, analysed the 30 untreated and the 23 treated patients separately. From 18 of the 30 untreated patients serum samples were accessible also at least 6 months after having started UDCA-treatment.

In eight patients orthotopic liver transplantation (OLT) had to be performed in the course of the disease. Observation period after OLT ranged from one month to 20 years. Three of these patients were pANCA positive before OLT.

Peripheral blood mononuclear cells (PBMC) were obtained from 33 of the 53 PSC-patients. All of them were already under UDCA-treatment; 17 suffered from IBD, 6 had further autoimmune disorders (Additional file 2).

Furthermore, sera from 60 untreated patients with AIH (females *n* = 41, males *n* = 19; mean age 53 years) and 95 untreated patients with PBC (females *n* = 81, males *n* = 14; mean age 56 years) were analysed. All patients with AIH had a score of at least 15 according to the criteria of the Autoimmune Hepatitis Study Group [16]. PBC patients were selected according to their reactivity with subunits of the 2-oxoacid dehydrogenase complex (OCD; M2-antigen), i.e. 50 were anti-M2/ODC positive, 45 were negative but had antibodies to nuclear dots (sp100), nuclear membrane (gp210) or centromeres [17].

Sera from 34 patients with alcoholic liver disease (ALD; females *n* = 9, males *n* = 25; mean age 54 years) and from 120 patients with viral hepatitis (hepatitis C, *n* = 92, hepatitis B, *n* = 28; females *n* = 42, males *n* = 78, mean age 41 years) were used as controls.

All patients with chronic liver diseases been seen by one of the two authors CPB or CW.

Moreover, sera from 43 patients with ulcerative colitis (UC; females *n* = 16, males *n* = 27; mean age 41 years) and 44 patients with Crohn’s disease (females *n* = 23, males *n* = 21; mean age 40 years; all histologically proven; kindly provided by Dr. A. Raible, Department of Internal Medicine I, Tuebingen) as well as from 26 patients with inflammatory connective tissue diseases (CTD; collagen disorders *n* = 19, rheumatoid arthritis n = 7; females *n* = 24, males n = 2; mean age 50 years; all seen by one of the authors [RK]), and 50 healthy donors (kindly provided by Dr. D. Wernet, Department of Transfusion Medicine, Tuebingen; females *n* = 38, males *n* = 12; mean age 44 years, range 23-59 years) were investigated.

The study had been approved by the local ethical committee of the Medical Faculty, University of Tuebingen (project number 076/2012BO1). Informed written consent was obtained from each patient included into the study, and the study protocol conforms to the ethical guidelines of the 1975 Declaration of Helsinki.

### Antigens

#### Generation of recombinant SO proteins in bacteria (*E. coli*)

Recombinant human full-length SO (SO-fl; amino acid [aa] 1-488) was generated in *E.coli* as described [11]. For cloning and expression of the SO-subunits the same procedure was used. The cytochrome b5-like heme/steroid binding domain of SO (SO-I;aa1-123) was generated with oligonucleotide primers covering bp3-543 (forward:5´-GGGACCCTATTAGGTCTCGGTG-3′, reverse:5´-TACAGGATCATCAGCATAAGG-3′), the oxidoreductase molybdopterin cofactor binding domain (SO-II; aa124-382) using primers covering bp544-1317 (forward:5´-CGTCACCCAGCCCTGAAGG-3′, reverse:5´-TTCTACAGTCTCTCCATCCCG-3′), the molybdopterin cofactor oxidoreductase dimerization domain (SO-III; aa310-488) with primers covering bp1102-1638 (forward:5´-GTGGTTCCTGGAGTGGTGG-3′, reverse:5´-TGGGGAGACATAGACATGGAC-3′) of the precursor form. The DNA-sequences of the plasmid constructs were verified by sequencing (Eurofins Genomics, Ebersberg, Germany).

Identity and integrity of the recombinant proteins SO-fl, SO-I, SO-II, and SO-III were confirmed by matrix-assisted laser desorption/ionization time of flight mass spectrometry (MALDI-TOF; kindly performed by Dr. S. Stevanovic, Department of Immunology, University of Tuebingen, Germany).

#### Purification of recombinant SO-related proteins

**Fig. 1:**
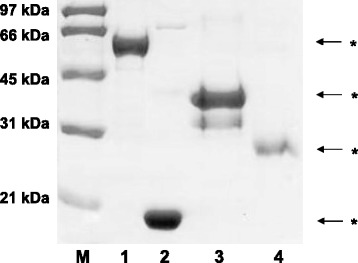
Demonstration of recombinant SO proteins by SDS-PAGE (10%; Coomassie-blue staining). M = molecular weight marker; 1 = SO-fl at 60 kDa; 2 = SO-I at 17 kDa; 3 = SO-II at 32 kDa; 4 = SO-III at 23 kDa. * = bands were excised and confirmed to correspond to SO by MALDI-TOF mass spectrometry

In order to obtain highly purified SO-proteins, SDS-electrophoresis was performed and the specific bands were excised after Coomassie blue-staining (Fig. 1) [11]. All recombinant proteins and subsequent preparations were shown to be negative for endotoxins by the limulus amebocyte lysate test (Pyrogent® 5000; Lonza, Walkersville, MD).

#### Synthetic SO-peptides

Twenty-nine synthetic peptides, spanning the entire length of human SO, were constructed according to the reported sequence (Biotrend, Cologne, Germany). They consisted of 25aa residues, with an overlap of eight aa each (Additional file 3). Purity was > 80% as verified by high performance liquid chromatography.

### Detection of autoantibodies

*Immunofluorescence test (IFT)* for the demonstration of different AIH- and PBC-related autoantibodies and pANCA, the *ELISA* for the detection of antibodies to recombinant SO-proteins and SO-peptides, as well as *SDS-polyacrylamide gel electrophoresis (PAGE) and Western blotting (WB)* were performed as described [11, 18].

For ELISA, patients’ sera were diluted 1:400. Antigen concentrations of 0.4 μg/ml (SO-fl and SO-II), 1.6 μg/ml (SO-I), 3 μg/ml (SO-III), and 50 μg/ml (synthetic SO-peptides) were applied. Optimal antigen- and serum-concentrations had been evaluated by serial dilution prior to this study.

In each assay three sera with high, medium and low anti-SO-reactivity as well as a negative serum were tested for the calculation of a standard curve to which all test samples were referred. Antibody activities are given as absorbance multiplied by 1000. Sera from 50 healthy individuals were used to determine cut-off values [11].

### Cellular immunoreactivity

#### PBMC cultures

Isolation of PBMC from heparinised blood was performed according to standard procedures [19, 20]. They were adjusted to one million cells/ml in RPMI 1640 medium containing gentamycin and 25% autologous serum and then incubated with or without antigens at 37 °C in a humidified atmosphere containing 5% CO_2_.

The SO-related antigens SO-fl, SO-I, SO-II, and SO-III were added to the PBMC in different concentrations ranging from 5 to 0.005 μg/ml (5, 0.5, 0.05, 0.005 μg/ml) in order to obtain the optimal ratio between specific immunocompetent cells and antigen concentrations for each patients’ PBMC. For statistical analysis the concentrations with the strongest effect on the PBMC were selected.

Prior to the experiments kinetics had been performed to obtain the optimal time points for the assays. For the determination of the early activation marker CD69 by flow cytometry PBMC were incubated with the different antigens for 24 h, for the analysis of cell proliferation and release of cytokines into the supernatant for seven days [19, 20].

#### Proliferation assay

Proliferation of PBMC was determined by thymidine-uptake according to standard procedures [19, 20]. Results are given as counts per minute (cpm). Mean values of four fold tests were calculated and used for statistical analysis.

#### Flow cytometry

For the determination of activated immune cells by flow cytometry, the PBMC were incubated with or without the antigens in different concentrations for 24 h. The activation marker CD69 was determined on T-, B-, and natural killer (NK)-cells as described using FastImmune™CD4/CD69/CD3,-CD8/CD69/CD3,-CD19/CD69/CD45, and -CD56/CD69/CD45 (Becton-Dickinson, San Jose, CA) [19, 20]. An IgG isotype control for each antibody (BD Biosciences Pharmingen, San Diego, CA) was used to set quadrants, and at least 10,000 cells were counted. Quadrants were set based upon the isotype controls for each antibody. Percentage of CD69 expressing cells of each cell type (CD4+/CD3+; CD8+/CD3+; CD19+/CD45+; CD56+/CD45+) was calculated.

#### Cytokine assay

Cytokines (interleukin [IL]-1,-5,-6,-10,-13, interferon[IFN]-γ tumour necrosis factor[TNF]α and –β) were determined in the PBMC supernatants after culturing them with the antigens in different concentrations for 7 days as recently described using antibody pairs and recombinant cytokines as standards (Pharmingen, San Diego, CA, USA) [19, 20].

Cut off values were 100 pg/ml for TNFβ and IL-17, 150 pg/ml for IL-10, 250 pg/ml for IL-6, 300 pg/ml for IL-13 and TNFα, 500 pg/ml for IL-1 and IL-5, and 1300 pg/ml for IFNγ.

#### Cell viability

Viability and function of the PBMC was evaluated by incubating them in parallel with poke weed mitogen (PWM; 40 μg/ml, Biochrom AG, Berlin, Germany) [19, 20]; activation of subpopulations was proven by incubation with the recall-antigens Bacillus Calmette-Guérin (BCG), purified protein derivative (PPD), and tetanus toxoid (TT) [19, 20]. PBMC which were not activated by any of these mitogens/antigens were omitted from further analyses.

### Statistics

For statistical analysis the Graph Pad Prism (version 4.03) and SPSS (version 21.0) programs were applied. The non-parametric Mann-Whitney test was used for unpaired data, the non-parametric Wilcoxon matched pairs test for paired data, the Fisher’s exact test for comparison of prevalence of anti-SO antibodies, and the Pearson product-moment correlation coefficient for determination of correlations between pANCA and anti-SO antibodies. *P*-values ≤0.05 were defined as significance levels. Significant data were further analysed by post hoc-tests.

Furthermore, receiver-operating curves (ROC) were calculated to confirm the cut off-levels and to evaluate differences between antibody reactivity in PSC-patients and patients with other disorders.

## Results

### Expression of SO in *E. coli*

The elution steps after expression of SO-fl, SO-I, SO-II or SO-III in *E.coli* were applied to SDS-electrophoresis. Bands at 60 k Dalton (kDa) for SO-fl, at 17 kDa for SO-I, at 32 kDa for SO-II, and at 23 kDa for SO-III were observed after protein staining (Fig. 1). The identity of these determinants with the respective SO-subunits was verified by MALDI-TOF spectrometry. In further investigations these proteins were used as recombinant SO-related antigen fractions.

### Identification of SO-related determinants by WB

By WB, the monoclonal antibody recognised the 60 kDa band of SO-fl and the 17 kDa band of SO-I. With patients’ sera different reaction patterns were observed reacting with at least one of the SO-related antigens. Sera from healthy individuals were negative (data not shown).

### Analysis of the humoral immunoreactivity towards SO-proteins

In order to avoid bacterial contaminations in the recombinant SO-fractions, the respective SO-bands (Fig. 1) were eluted from the SDS-gels and applied as highly purified antigens in the ELISA. The *prevalence* of IgG-antibodies to SO-fl and SO-I (aa1-123) and of IgM-antibodies to SO-fl, SO-II (aa124-382) and SO-III (aa310-488) was significantly higher in patients with PSC as compared to patients with other diseases including IBD (Table 1).

**Table 1:** Prevalence of anti-SO antibodies of the IgG- and IgM-type in sera from patients with untreated PSC as compared to controls

Diagnosis	No. patients	Antibodies to			
		SO-fl	SO-I	SO-II	SO-III
		IgG-type: number (%) positive			
Untreated PSC	30	13 (43)	7 (23)	6 (20)	3 (10)
PBC	95	0 ^*)^	0 ^*)^	0 ^*)^	2 (2)
AIH	60	4 (7) ^*)^	3 (5) ^*)^	4 (7) ^*)^	6 (10)
ALD	34	2 (6) ^*)^	2 (6) ^*)^	2 (6)	7 (20)
Hepatitis B or C	120	2 (2) ^*)^	0 ^*)^	2 (2) ^*)^	9 (7)
Ulcerative colitis	43	4 (9) ^*)^	2 (5) ^*)^	3 (7)	3 (7)
Crohn’s disease	44	5 (11) ^*)^	4 (9)	4 (9)	4 (9)
CTD	26	0 ^*)^	0 ^*)^	2 (8)	3 (11)
Blood donors	50	3 (6) ^*)^	3 (6) ^*)^	3 (6)	2 (4)
		IgM-type: number (%) positive			
Untreated PSC	30	7 (23)	3 (10)	10 (33)	14 (47)
PBC	95	0 ^*)^	0 ^*)^	0 ^*)^	0 ^*)^
AIH	60	2 (3) ^*)^	1 (2)	2 (3) ^*)^	1 (2) ^*)^
ALD	34	2 (6) ^*)^	0	1 (3) ^*)^	1 (3) ^*)^
Hepatitis B or C	120	2 (2) ^*)^	2 (2)	3 (2) ^*)^	2 (2) ^*)^
Ulcerative colitis	43	2 (5) ^*)^	2 (5)	1 (2) ^*)^	2 (5) ^*)^
Crohn’s disease	44	1 (2) ^*)^	1 (2)	5 (11) ^*)^	4 (9) ^*)^
CTD	26	0 ^**)^	0	0 ^**)^	0 ^**)^
Blood donors	50	3 (6) ^*)^	3 (6)	1 (2) ^*)^	2 (4) ^*)^

^*^significantly lower as compared to patients with untreated PSC, *p* < 0.05

**Fig. 2:**
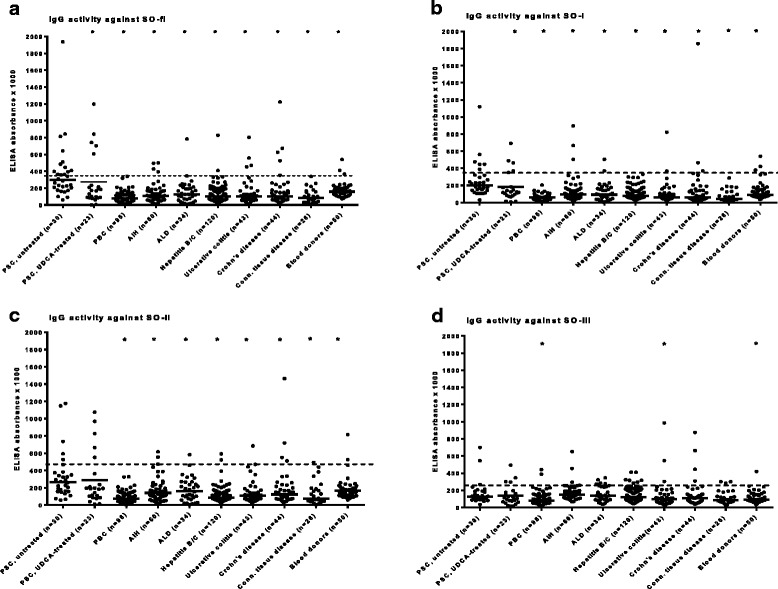
Activity of anti-SO-antibodies of the IgG-type towards SO-fl (**a**), SO-I (cytochrome b5-like heme/steroid binding domain) (**b**), SO-II (oxidoreductase molybdopterin cofactor binding domain) (**c**) and SO-III (molybdopterin cofactor oxidoreductase dimerization domain) (**d**) in sera from patients with different hepatic and non-hepatic disorders as determined by ELISA. Individual values (•) and median (**―**) are given. ^_^ - -: threshold for positivity. * significant as compared to untreated PSC patients; *p* < 0.05 (with post-hoc tests Tukey and Bonferroni)

Furthermore, the IgG-antibody *reactivity* against SO-fl, SO-I and SO-II was significantly higher in the untreated PSC-patients as compared to the other groups of patients and blood donors (Fig. 2a-c). Again, reactivity with SO-III was less specific for PSC because only patients with PBC, CTD or blood donors showed a significant decrease in IgG antibody activities as compared to PSC patients (Fig. 2d). Calculation of sensitivity, specificity and accuracy revealed values of 43%, 96%, and 93% for antibodies to SO-fl, 23%, 97% and 93% for antibodies to SO-I, 20%, 96% and 91% for antibodies to SO-II, and 10%, 92% and 87% for antibodies to SO-III.

There was a significant correlation between antibody reactivity towards SO-fl and that towards SO-I, -II, and –III (*p* < 0.05; data not shown).

Similar data were obtained for IgM-antibody reactivity.

The PSC-specificity of IgG- and IgM-antibodies to SO-fl, SO-I and SO-II was also proven by ROC-analysis calculating the areas under the curves (AUC) (Additional file 4); for SO-fl, SO-I and SO-II PSC-sera differed significantly from all other groups. For SO-III, PSC-patients differed only from patients with PBC, IBD and from healthy controls.

Within the PSC-group there was no significant difference in SO-reactivity between those with (*n* = 15) or without IBD (n = 15) (Additional file 5), but both groups showed higher anti-SO-reactivity towards SO-fl, SO-I and SO-II than patients with IBD alone.

Comparing UDCA-treated (*n* = 23) and untreated PSC-patients (*n* = 30), in the treated patients IgG- (and IgM-) antibody reactivity against SO-fl and SO-I was significantly reduced (Fig. 2). Also in the 18 patients who were untreated and of whom sera were available in the course of the disease during therapy with UDCA, a significant decrease of antibody reactivity was observed during therapy (*p* < 0.01); however, this decrease was only observed after treatment for more than 2 years, but then it persisted for an observation period of more than 15 years. Reappearance of the antibodies was not observed. Laboratory parameters (alkaline phosphatase [AP], gamma-glutamyl-transferase [γGT], alanine aminotransferase [ALAT], aspartate aminotransferase [ASAT]) significantly decreased during UDCA-treatment (Additional file 2), but there was no correlation with any anti-SO-antibody reactivity, neither before nor during UDCA-therapy (not shown).

Moreover, pANCA and anti-SO antibodies did not correlate as already shown in our previous study [11]. Thus, one of the 30 untreated PSC patients (3%) had both, pANCA and anti-SO-fl antibodies; 12 (40%) had only anti-SO-fl antibodies and nine (30%) only pANCA.

All eight PSC-patients in whom OLT had to be performed were anti-SO negative (for all SO-subunits) before OLT due to UDCA-treatment. In none of them anti-SO antibodies appeared after OLT even for an observation period of up to 20 years. In contrast, in all three patients being pANCA positive before OLT, these antibodies persisted after OLT.

**Fig. 3:**
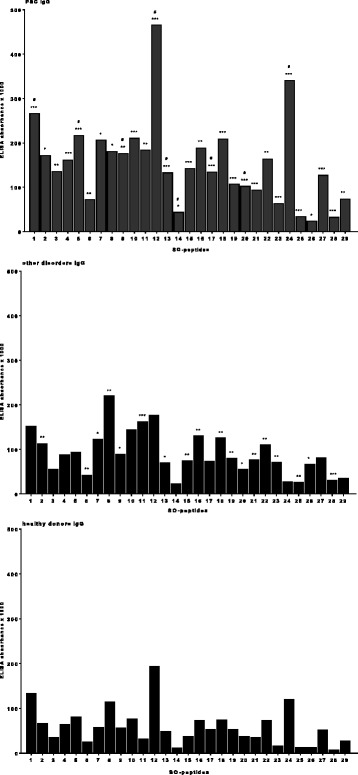
IgG-reactivity of sera from 11 anti-SO positive PSC-patients, 10 patients with other disorders, and 10 healthy controls with 29 SO-peptides (25aa, 8aa overlapping) in the ELISA. The median is given. Significant as compared to the blood donors: * p < 0.05; ** *p* < 0.01; *** *p* < 0.001

In order to define immuno-dominant epitopes of SO, sera from 11 PSC patients being anti-SO positive were tested against 29 peptides with 25aa by ELISA. Reactivity to all peptides was significantly higher in PSC patients than in healthy controls, however also sera from patients with other disorders had higher antibody reactivity to most peptides than healthy control (Fig. 3). In PSC-patients, the strongest antibody reactivity was observed towards peptides 12 (aa188-212, SO-II) and 24 (aa392-416, SO-III) (see also Additional file 1), and for these peptides as well as peptides 1 (aa1-25, SO-I), 5 (aa69-93, SO-II), 9 (aa137-161), 13 (aa205-229, SO-II), 14 (aa222-246, SO-II), 17 (aa273-297) and 20 (aa324-348) sera from PSC patients showed also higher reactivity than sera from patients with other disorders.

For IgM-antibodies a rather heterogeneous reaction pattern was observed (not shown).

### Analysis of the cellular immune-reactivity towards SO-proteins

**Fig. 4:**
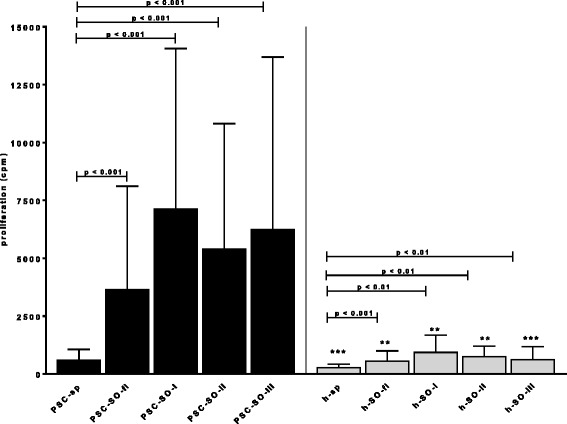
Proliferative response of peripheral blood mononuclear cells from 33 PSC patients and 10 healthy individuals towards the four SO-proteins SO-fl, SO-I, SO-II, SO-III as measured by lymphocyte transformation test (cpm = counts per minute). Sp = spontaneous proliferation without antigen. Significance levels as compared to the spontaneous proliferation are indicated. Significance as compared to PSC-patients: * *p* < 0.05; ** *p* < 0.01; *** *p* < 0.001

All four SO-related antigens strongly enhanced the *proliferative response* of PBMC from PSC-patients in a dose-depended manner being strongest at the highest concentration of 5 μg/ml. They also induced proliferation of PBMC from healthy donors, but to much less extent as compared to the PSC-PBMC (Fig. 4).

**Fig. 5:**
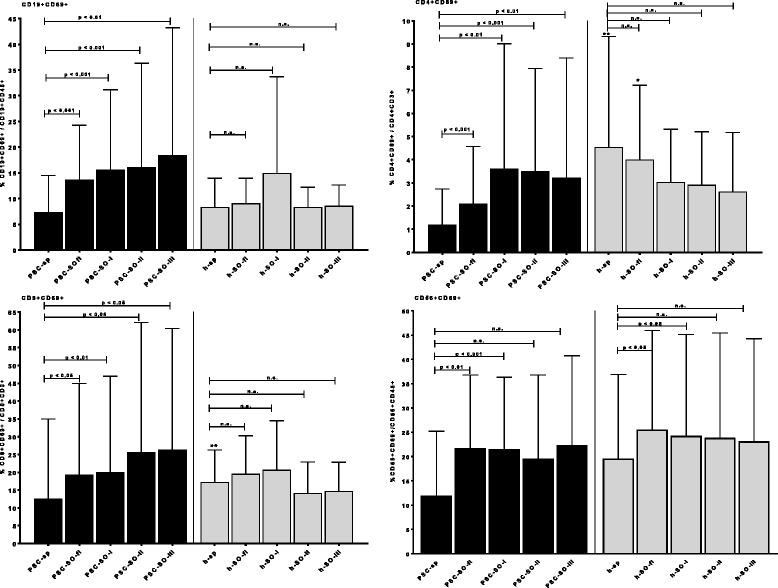
Influence of the four SO-antigens SO-fl, SO-I, SO-II, and SO-III on the expression of the activation marker CD69 by CD19+ B-cells, CD4+ helper- and CD8+ cytotoxic T-cells and CD56+ NK-cells from 20 PSC patients and 10 healthy individuals as measured by flow cytometry. The percentage of activated (CD69+) CD19+ B-cells, CD4+ or CD8+ T-cells of all CD19+ B-, CD4+ or CD8+ T-cells is given. sp. = spontaneous CD69 expression without antigen; significant as compared to the spontaneous CD69-expression: * *p* < 0.05; ** *p* < 0.01; *** *p* < 0.001; # significant as compared to the reaction of healthy controls (*p* < 0.05)

Moreover, incubation with SO-related antigens strongly enhanced the expression of the *activation marker CD69* by CD19+/CD45+ B-cells from PSC-patients but not from healthy controls (Fig. 5). Spontaneous CD69 expression by CD4+ and CD8+ T-cells was significantly lower in PSC-patients (median 0.70% and 4.25%) than in the control group (median 2.32% and 14.32%). Again, there was a statistically significant increase of CD69-expression after incubation of PSC-PBMC with SO-fl, SO-I, -II, and-III, while the activation of both T-cell subpopulations from healthy donors was rather reduced (although statistically not significant) (Fig. 5). NK-cells were activated in both groups only by SO-fl and SO-I.

**Fig. 6:**
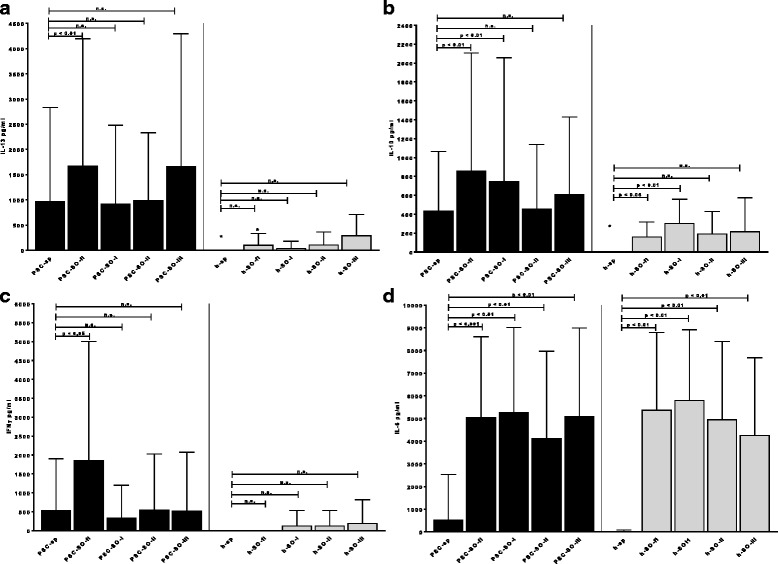
IL-13 (**a**), IL-10 (**b**), IFNγ (**c**), and IL-6-production (**d**) by PBMC from 20 PSC-patients and 10 healthy controls after incubation with four SO-related antigens. sp. = spontaneous cytokine release without antigen; significant as compared to the spontaneous cytokine release: * *p* < 0.05; ** *p* < 0.01; *** *p* < 0.001; # significant as compared to the reaction of healthy controls (*p* < 0.05)

Analysis of cytokine production revealed that PBMC from PSC-patients produced already spontaneously significantly higher amounts of IL-13 and IL-10 than that from healthy controls (Fig. 6a, b); IL-13 production was significantly enhanced by incubation of the PBMC from PSC patients with SO-fl but not the other SO-antigens. Moreover, incubation of PBMC from PSC patients with SO-fl and SO-I lead to a significant increase of IL-10 production. The same effect – but to less extent - was observed for PBMC from healthy individuals (Fig. 6b).

Also, IFNγ-production was higher by PBMC from PSC patients than from controls, but the difference was statistically not significant due to the large standard deviations (Fig. 6c). Incubation with SO-fl but not the other SO-proteins significantly enhanced the IFNγ-secretion by PBMC from PSC patients but not from controls.

Spontaneous IL-6-production by PBMC did not differ between PSC-patients and controls, but in both groups incubation with the four SO-proteins lead to a strong and significant increase of IL-6-secretion (Fig. 6d).

There was no effect of the SO-related antigens on the secretion of IL-5, TNFβ, IL-1α, IL-17 or TNFα by PBMC from PSC-patients and controls (data not shown).

A correlation coefficient of *r* = 0.83 was calculated between anti-SO-fl-antibody reactivity and SO-fl-induced CD69-expression by B-cells. A similar correlation (*r* = 0.80) was observed between anti-SO-III-antibody reactivity and SO-III induced B-cell activation. There was no relation between antibody-reactivity and other cellular reactivities towards the SO-antigens (data not shown).

Moreover, there was no significant difference in any cellular reactivity between PSC-patients with or without IBD (data not shown), which may be, however, due to the low number of patients.

## Discussion

In this study we found a strong humoral and cellular immune reactivity in PSC-patients towards SO previously suggested being a relevant autoantigen in PSC [11]. Included in the analysis were also its three major domains, the cytochrome b5 heme-binding (SO-I)-, the molybdenum-pterin (SO-II)- and the immunoglobulin-like domain (SO-III). Antibodies to SO-fl, to SO-I, and to less extent to SO-II were specific for PSC, while SO-III was recognised also by sera from patients with other liver disorders. The antibodies were of the IgG- and IgM-type, but the IgG-antibodies seem to be more relevant.

Anti-SO-fl antibodies were present in 43% of untreated PSC-patients, 42% had pANCA. We confirmed our previous data indicating that there was no correlation or cross-reactivity between both antibody types and that anti-SO-antibodies are more specific for PSC than pANCA [11]. Combining both antibodies, i.e. pANCA and anti-SO, only 8 (27%) of our untreated PSC patients were still antibody-negative. Whether the presence of anti-SO antibodies in the few patients with other liver disorders and IBD may be indicative for subclinical PSC and especially in case of AIH for an overlap syndrome with PSC [21] has to be evaluated in the further course of the disease.

In contrast to pANCA, prevalence and reactivity of anti-SO antibodies especially to SO-fl, SO-I and SO-II was significantly lower in patients with IBD than with PSC. Furthermore, we confirmed our previous observation that anti-SO-antibodies seem to decrease during UDCA-treatment while the pANCA remain unaffected, and this effect was independently whether the antibodies were directed against SO-fl or its three subunits. Since it has been shown that hydrophobic bile acids enhance SO-enzyme-activity and that the enzyme is released into the cytosol during apoptosis [22, 23], one could hypothesise that accumulation of these pro-apoptotic toxic bile acids in PSC may result in an altered presentation of the enzyme or the formation of a neo-antigen which can then be recognised by immunocompetent cells. Treatment with the hydrophilic UDCA, which has anti-apoptotic properties, may then reduce SO-expression and SO-related autoimmunity.

Interestingly, the anti-SO antibodies, which were negative in all eight patients in whom OLT had to be performed, probably due to UDCA-treatment, remained negative after OLT even for a long observation period. However, in all the patients UDCA-therapy was continued after OLT. In contrast, in the three patients being pANCA positive before OLT, the antibodies persisted despite immunosuppressive and UDCA treatment after OLT.

In order to define immuno-dominant epitopes for anti-SO-antibodies, sera were tested against 29 peptides representing the entire SO protein. However, no distinct immuno-dominant region specific for PSC could be identified. The PSC-specific reactivity towards SO-fl and SO-I may be, therefore, rather related to conformational epitopes. The strongest reactivity of PSC-sera was observed towards the two peptides 12(aa188-212) and 24(aa392-416) located in the molybdenum-pterin- and the C-terminal immunoglobulin-like dimerization domain, respectively, but these peptides were also recognised – although to less extent – by sera from anti-SO-negative patients with other disorders and healthy controls. Interestingly, the peptide aa188-212 comprises the binding sites for molybdenum pterin in position aa 207. Recent data suggest that the binding of molybdenum cofactor (Moco) is essential for the unidirectional translocation of SO into the mitochondria [24]. Accumulation of SO in the cytosol could make the protein more accessible for the immune system. Performing Basic Local Alignment Search Tool (BLAST) analysis, these two peptides 12 and 24 showed high similarity with SO from various bacteria - as expected because it is an archaic enzyme - but also some nematodes. Furthermore, SO occurs in plants and is highly similar to another enzyme, nitrate reductase. The Moco-binding domain belongs to ubiquitous super families including a very diverse range of proteins [25, 26]. One could, therefore, speculate that anti-SO antibodies are induced by molecular mimicry. This may also explain the presence of antibodies towards these peptides in patients with other disorders and even healthy controls suggesting the existence of a natural humoral autoreactivity towards SO-peptides.

To the contrary, we did not find a significant natural autoreactivity to SO on the *cellular level* in healthy individuals. This is in contrast to other autoimmune diseases [27–30]. Thus, PBMC from healthy individuals showed – in contrast to PBMC from PSC-patients – only a slight proliferative response towards SO-antigens. Moreover, incubation of PBMC from PSC-patients with SO-antigens strongly enhanced the activation, i.e. CD69-expression, of B-, CD4 + − and CD8+ T-cells from PSC-patients but not from healthy controls. Also cytokine production –except for IL-6 (see below) – was only marginally induced in PBMC from healthy individuals as compared to the PSC-patients. SO may, therefore, lead to T-cell-dependent B-cell activation in PSC. The state of activation of T-cells was in PSC even significantly lower than in the healthy individuals, and in the latter group the SO-antigens rather reduced the CD69 expressing T-cells; i.e. SO may have an inhibitory effect on pre-activated T-cells. In vivo it has been shown that T-cells may be rendered anergic through continuous stimulation with antigen and can become negative regulators of immune responses by releasing IL-10 [31]. This fits to our finding of a strong IL-10-production by PBCM from PSC-patients which was even enhanced by incubation with SO. On the other hand it is known that IL-10 can be produced also by B-cells, especially regulatory B cells [32]. It is well-established that autoantigen-specific B-cells, when present in the repertoire, are the first subset of antigen presenting cells to capture and present self-proteins for activating T-cells [33]. B-cells producing IL-10 have been shown to down regulate TH1-reactivity and ameliorate TH1-diseases [32], hereby increasing TH2-reactivity. Due to limited numbers of PBMC we could not analyse whether in our patients IL-10 was, indeed, produced by regulatory B- or T-cells. However, the strong release of IL-13 and IL-10 by PBMC from PSC-patients – a phenomenon which we did not observe with PBMC from PBC-patients (own unpublished observation) – also argues in favour of TH2-mechanisms involved in PSC as already shown for autoimmune pancreato-cholangitis [34]. TH2-cytokines, and especially IL-13, are known to be under certain conditions pro-fibrotic factors and to promote fibrocyte differentiation [35–38]; this may be related to the fibrotic changes in the liver in PSC. Indeed, antagonism of IL-13 signalling ameliorates fibrosis [39].

Interestingly, SO strongly enhanced IL-6-production by PBMC not only from PSC patients but also from healthy donors. A non-specific effect due to probable contamination with endotoxins could be excluded with high possibility by a negative Limulus test in these fractions. Within PBMC macrophages and B-cells are the strongest IL-6-producers. A release by macrophages in our setting seems unlikely due to the lack of production of other pro-inflammatory cytokines (TNFα or IL-1). Therefore, a release by B-cells could be taken into consideration as already reported for the autoantigen thyroglobulin [40, 41]. This SO-induced increase of IL-6 production may explain the strongly enhanced activation/CD69-expression by NK-cells in both groups of probands since it has been shown that IL-6 increases NK-cell activity [42].

We are aware that the cellular data presented here for PSC-patients and healthy controls have to be deepened by further studies; thus all PSC-patients in whom these analyses could be performed were already under UDCA-therapy. Moreover, due to the limited amounts of PBMC available from each PSC-patient we restricted our analyses in the present study to parameters which have been shown in previous investigations on cellular immunity to give reliable and significant results [19, 20, 43, 44], but further markers for immunocompetent cells might be of interest. Also, the analysis of PBMC from patients with other liver disorders with respect to their SO-reactivity will be important; our preliminary results indicate that also PBMC from some patients with ALD and AIH – but not PBC – show reactivity to SO-antigens, which seem to differ, however, with respect to cytokine-production and lymphocyte-activation from that in PSC-patients (own unpublished observation).

## Conclusions

Antibodies to sulphite oxidase, especially to the N-terminal cytochrome b5-like heme/steroid- and the oxidoreductase molybdopterin cofactor binding domain, are rather specific for PSC and can be detected in about 43% of patients. They may be, therefore, helpful to improve the serological diagnosis of PSC and probably also of overlap syndromes as for instance AIH/PSC. The antibodies may be directed preferentially against conformational epitopes, although two major immuno-dominant regions (aa188-212) and (aa392-416) located in the molybdenum-pterin- and the C-terminal immunoglobulin-like dimerization domain could be identified; these are, however, recognised also by sera from controls indicating that the antibodies may belong to the pool of natural autoantibodies.

Peripheral blood mononuclear cells (PBMC) from PSC patients produced already spontaneously mainly the pro-fibrotic TH2-cytokine IL-13 as well as IL-10. Incubation of PBMC from PSC patients with SO-related antigens induced an activation of B- but hardly T-cells and enhanced the type-2-cytokine pattern. Until now, there is only little known about the role of SO in the liver in general and especially in cholangiocytes; in this respect, the induction of pro-fibrotic cytokines by PBMC from PSC as described here is of interest. Further studies on the role of SO in PSC might, therefore, help to elucidate its relationship to the (aetio?)pathogenesis of the disease.

## Additional files

Additional file 1: Schematic presentation of the enzyme sulphite oxidase (SO) with its different domains, and the immuno-dominant epitopes recognised by PSC sera (aa = amino acid; mo = molybdenum binding site). (PDF 165 kb)

Additional file 2: Clinical data and laboratory parameters before and during UDCA-treatment and availability of serum and peripheral blood mononuclear cells in 53 PSC patients included in the study. (DOCX 30 kb)

Additional file 3: Overlapping synthetic peptides of human SO protein chain. (PDF 193 kb)

Additional file 4: Receiver operating curve (ROC) analysis for IgG-antibodies to the four SO-proteins comparing sera from PSC patients with those from healthy donors, PBC, AIH, alcoholic liver disease (ALD), viral hepatitis, ulcerative colitis (CU), Crohn disease (CD), and collagen disorders. (PDF 257 kb)

Additional file 5: Activity of IgG-antibodies against the four SO-proteins in sera from untreated PSC-patients without and with IBD as well as in patients with pure IBD as determined by ELISA. Individual values (•) and median (**―**) are given. *p* < 0.05 (with post-hoc tests Tukey and Bonferroni). (PDF 164 kb)

## Abbreviations

AIH: Autoimmune hepatitis
ALD: Alcoholic liver disease
AUC: Area under the curve
cpm: Counts per minute
CTD: Connective tissue disease
DNA: Deoxyribonucleic acid
ELISA: Enzyme-linked immunosorbent assay
GM-CSF: Granulocyte-macrophage-colony-stimulating factor
HRP: Horse radish peroxidase
IBD: Inflammatory bowel disease
IFN: Interferon
IFT: Immunofluorescence test
IL: Interleukin
kDa: Kilo-Dalton
MALDI-TOF: Matrix-assisted laser desorption/ionisation time of flight mass spectrometry
NK: Natural killer
NTA: Nitrilotriacetic acid
OCD: 2-oxo-acid-dehydrogenase complex
OLT: Orthotopic liver transplantation
pANCA: Antibodies to neutrophilic cytoplasmic antigens of the perinuclear type
PBC: Primary biliary cholangitis
PBMC: Peripheral blood mononuclear cells
PWM: Poke weed mitogen
ROC: Receiver-operating curves
SD: Standard deviation
SDS: Sodium-dodecyl-sulfate
SO: Sulphite oxidase
TNF: Tumour necrosis factor
UDCA: Ursodeoxycholic acid

## References

[1] Ponsioen CY (2012). Recent insights in primary sclerosing cholangitis. J Dig Dis.

[2] Chapman R, Cullen S (2008). Etiopathogenesis of primary sclerosing cholangitis. World J Gastroenterol.

[3] Pollheimer MJ, Halilbasic E, Fickert P, Trauner M (2011). Pathogenesis of primary sclerosing cholangitis. Best Pract Res Clin Gastroenterol.

[4] Trivedi PJ, Chapman RW (2012). PSC, AIH and overlap syndrome in inflammatory bowel disease. Clin Res Hepatol Gastroenterol.

[5] Klein R, Eisenburg J, Weber P, Seibold F, Berg PA (1991). Significance and specificity of antibodies to neutrophils detected by western blotting for the serological diagnosis of primary sclerosing cholangitis. Hepatology.

[6] Terjung B, Spengler U (2009). Atypical p-ANCA in PSC and AIH: a hint toward a "leaky gut"?. Clin Rev Allergy Immunol.

[7] Terjung B, Sohne J, Lechtenberg B, Gottwein J, Muennich M, Herzog V, Mahler M, Sauerbruch T, Spengler U (2010). P-ANCAs in autoimmune liver disorders recognise human beta-tubulin isotype 5 and cross-react with microbial protein FtsZ. Gut.

[8] Bansi DS, Fleming KA, Chapman RW (1995). Antineutrophil cytoplasmic antibodies in autoimmune hepatitis. Gastroenterology.

[9] Prideaux L, De Cruz P, Ng SC, Kamm MA (2012). Serological antibodies in inflammatory bowel disease: a systematic review. Inflamm Bowel Dis.

[10] Dobric S, Popovic D, Nikolic M, Andrejevic S, Spuran M, Bonaci-Nikolic B (2011). Anti-neutrophil cytoplasmic antibodies (ANCA) specific for one or several antigens: useful markers for subtypes of ulcerative colitis and associated primary sclerosing cholangitis. Clin Chem Lab Med.

[11] Preuss B, Berg C, Altenberend F, Gregor M, Stevanovic S, Klein R (2007). Demonstration of autoantibodies to recombinant human sulphite oxidase in patients with chronic liver disorders and analysis of their clinical relevance. Clin Exp Immunol.

[12] Klein R, Berg PA (1991). Anti-M4 antibodies in primary biliary cirrhosis react with sulphite oxidase, an enzyme of the mitochondrial inter-membrane space. Clin Exp Immunol.

[13] Feng C, Tollin G, Enemark JH (2007). Sulfite oxidizing enzymes. Biochim Biophys Acta.

[14] Kisker C, Schindelin H, Pacheco A, Wehbi WA, Garrett RM, Rajagopalan KV, Enemark JH, Rees DC (1997). Molecular basis of sulfite oxidase deficiency from the structure of sulfite oxidase. Cell.

[15] Johnson JL, Coyne KE, Garrett RM, Zabot MT, Dorche C, Kisker C, Rajagopalan KV (2002). Isolated sulfite oxidase deficiency: identification of 12 novel SUOX mutations in 10 patients. Hum Mutat.

[16] Alvarez F, Berg PA, Bianchi FB, Bianchi L, Burroughs AK, Cancado EL, Chapman RW, Cooksley WG, Czaja AJ, Desmet VJ (1999). International autoimmune hepatitis group report: review of criteria for diagnosis of autoimmune hepatitis. J Hepatol.

[17] Invernizzi P, Selmi C, Ranftler C, Podda M, Wesierska-Gadek J (2005). Antinuclear antibodies in primary biliary cirrhosis. Semin Liver Dis.

[18] Braun S, Berg C, Buck S, Gregor M, Klein R (2010). Catalytic domain of PDC-E2 contains epitopes recognized by antimitochondrial antibodies in primary biliary cirrhosis. World J Gastroenterol.

[19] Barth H, Berg PA, Klein R (2003). Methods for the in vitro determination of an individual disposition towards TH1- or TH2-reactivity by the application of appropriate stimulatory antigens. Clin Exp Immunol.

[20] Thaher F, Plankenhorn S, Klein R (2011). Differential effects of the tumor necrosis factor alpha-blocker infliximab and etanercept on immunocompetent cells in vitro. Int Immunopharmacol.

[21] Bogdanos DP, Mieli-Vergani G, Vergani D (2009). Autoantibodies and their antigens in autoimmune hepatitis. Semin Liver Dis.

[22] Joshi VC, Kurup CK, Ramasarma T (1969). The cryptic nature of hepatic microsomal sulphite oxidase. Biochem J.

[23] Lofrumento DD, La Piana G, Palmitessa V, Abbrescia DI, Lofrumento NE (2016). Stimulation by pro-apoptotic valinomycin of cytosolic NADH/cytochrome c electron transport pathway-effect of SH reagents. Int J Biochem Cell Biol.

[24] Klein JM, Schwarz G (2012). Cofactor-dependent maturation of mammalian sulfite oxidase links two mitochondrial import pathways. J Cell Sci.

[25] Mendel RR, Kruse T (2012). Cell biology of molybdenum in plants and humans. Biochim Biophys Acta.

[26] Schenkman JB, Jansson I (2003). The many roles of cytochrome b5. Pharmacol Ther.

[27] Scholz C, Patton KT, Anderson DE, Freeman GJ, Hafler DA (1998). Expansion of autoreactive T cells in multiple sclerosis is independent of exogenous B7 costimulation. J Immunol.

[28] Shimoda S, Ishikawa F, Kamihira T, Komori A, Niiro H, Baba E, Harada K, Isse K, Nakanuma Y, Ishibashi H (2006). Autoreactive T-cell responses in primary biliary cirrhosis are proinflammatory whereas those of controls are regulatory. Gastroenterology.

[29] Viglietta V, Kent SC, Orban T, Hafler DA (2002). GAD65-reactive T cells are activated in patients with autoimmune type 1a diabetes. J Clin Invest.

[30] Zou J, Hannier S, Cairns LS, Barker RN, Rees AJ, Turner AN, Phelps RG (2008). Healthy individuals have Goodpasture autoantigen-reactive T cells. J Am Soc Nephrol.

[31] Barrat FJ, Cua DJ, Boonstra A, Richards DF, Crain C, Savelkoul HF, de Waal-Malefyt R, Coffman RL, Hawrylowicz CM, O'Garra A (2002). In vitro generation of interleukin 10-producing regulatory CD4(+) T cells is induced by immunosuppressive drugs and inhibited by T helper type 1 (Th1)- and Th2-inducing cytokines. J Exp Med.

[32] Vadasz Z, Haj T, Kessel A, Toubi E (2013). B-regulatory cells in autoimmunity and immune mediated inflammation. FEBS Lett.

[33] Lund FE, Randall TD (2010). Effector and regulatory B cells: modulators of CD4+ T cell immunity. Nat Rev Immunol.

[34] Zen Y, Fujii T, Harada K, Kawano M, Yamada K, Takahira M, Nakanuma Y (2007). Th2 and regulatory immune reactions are increased in immunoglobin G4-related sclerosing pancreatitis and cholangitis. Hepatology.

[35] Liu L, Kou P, Zeng Q, Pei G, Li Y, Liang H, Xu G, Chen S (2012). CD4+ T lymphocytes, especially Th2 cells, contribute to the progress of renal fibrosis. Am J Nephrol.

[36] Oriente A, Fedarko NS, Pacocha SE, Huang SK, Lichtenstein LM, Essayan DM (2000). Interleukin-13 modulates collagen homeostasis in human skin and keloid fibroblasts. J Pharmacol Exp Ther.

[37] Saito A, Okazaki H, Sugawara I, Yamamoto K, Takizawa H (2003). Potential action of IL-4 and IL-13 as fibrogenic factors on lung fibroblasts in vitro. Int Arch Allergy Immunol.

[38] Shao DD, Suresh R, Vakil V, Gomer RH, Pilling D (2008). Pivotal advance: Th-1 cytokines inhibit, and Th-2 cytokines promote fibrocyte differentiation. J Leukoc Biol.

[39] Chiaramonte MG, Donaldson DD, Cheever AW, Wynn TA (1999). An IL-13 inhibitor blocks the development of hepatic fibrosis during a T-helper type 2-dominated inflammatory response. J Clin Invest.

[40] Langkjaer A, Kristensen B, Hansen BE, Schultz H, Hegedus L, Nielsen CH (2012). B-cell exposure to self-antigen induces IL-10 producing B cells as well as IL-6- and TNF-alpha-producing B-cell subsets in healthy humans. Clin Immunol.

[41] Nielsen CH, Brix TH, Leslie RG, Hegedus L (2009). A role for autoantibodies in enhancement of pro-inflammatory cytokine responses to a self-antigen, thyroid peroxidase. Clin Immunol.

[42] Cheekatla SS, Tripathi D, Venkatasubramanian S, Nathella PK, Paidipally P, Ishibashi M, Welch E, Tvinnereim AR, Ikebe M, Valluri VL (2016). NK-CD11c+ cell crosstalk in diabetes enhances IL-6-mediated inflammation during Mycobacterium tuberculosis infection. PLoS Pathog.

[43] Barth H, Klein K, Bortlein A, Guseo A, Berg PA, Wietholter H, Klein R (2002). Analysis of immunoregulatory T-helper cell subsets in patients with multiple sclerosis: relapsing-progressive course correlates with enhanced T H1, relapsing-remitting course with enhanced T H0 reactivity. J Neuroimmunol.

[44] Klein R, Buck S, Classen K, Rostock M, Huber R (2008). Enhanced in vitro activation of immunocompetent cells in healthy individuals being subcutaneously 'vaccinated' with placebo (physiological saline). Clin Immunol.

