# Supplementary material for: Sulphite oxidase (SO) – a mitochondrial autoantigen as target for humoral and cellular immune reactions in primary sclerosing cholangitis
Source: BMC Gastroenterol. 2018 May 2;18:58. doi: 10.1186/s12876-018-0787-x (PMC5932765; doi:10.1186/s12876-018-0787-x)
Supplement: Supplementary file 1 — Schematic presentation of the enzyme sulphite oxidase (SO) with its different domains, and the immuno-dominant epitopes recognised by PSC sera (aa = amino acid; mo = molybdenum binding site). (PDF 165 kb) [file 12876_2018_787_MOESM1_ESM.pdf]

Additional file 1

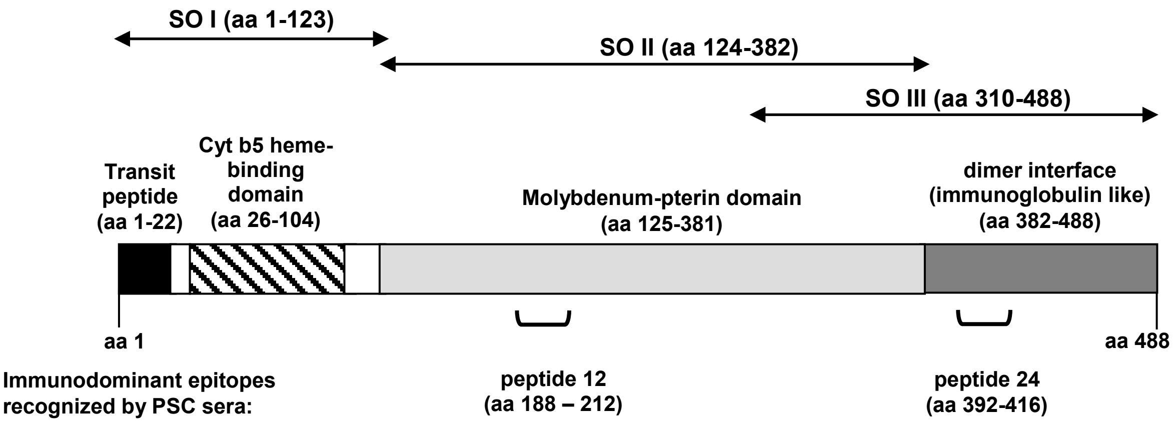

Schematic presentation of the enzyme sulfite oxidase (SO) with its different domains, and the immunodominant epitopes recognized by PSC sera (aa = amino acid; mo = molybdenum binding site)
