# Supplementary material for: Sulphite oxidase (SO) – a mitochondrial autoantigen as target for humoral and cellular immune reactions in primary sclerosing cholangitis
Source: BMC Gastroenterol. 2018 May 2;18:58. doi: 10.1186/s12876-018-0787-x (PMC5932765; doi:10.1186/s12876-018-0787-x)
Supplement: Supplementary file 2 — Clinical data and laboratory parameters before and during UDCA-treatment and availability of serum and peripheral blood mononuclear cells in 53 PSC patients included in the study. (DOCX 30 kb) [file 12876_2018_787_MOESM2_ESM.docx]

**Additional file 2 Clinical data and laboratory parameters before and during UDCA-treatment and availability of serum and peripheral blood mononuclear cells in 53 PSC patients included in the study**

| no | IBD | other AI  disease | PBMC  available | serum available | | AP (IU) ^a)^ | | γGT (IU) ^a)^ | | ASAT (IU) ^a)^ | | ALAT (IU) ^a)^ | | bilirubin (mg%) | | pANCA  rec titer |
| --- | --- | --- | --- | --- | --- | --- | --- | --- | --- | --- | --- | --- | --- | --- | --- | --- |
|  |  |  |  | before therapy | during therapy | before therapy | during therapy | before therapy | during therapy | before therapy | during therapy | before therapy | during therapy | before therapy | during therapy |  |
| 1 | UC |  | - | X | X | 1330 | 578 | 230 | 36 | 53 | 29 | 40 | 22 | 3,2 | 1,8 | 1280 |
| 2 | none | AIH | X | X | X | 900 | 148 | 411 | 54 | 56 | 26 | 73 | 11 | 1,1 | 1,7 | 0 |
| 3 | none | AIH | - | X | - | 400 | 71 | 255 | 41 | 85 | 9 | 250 | 7 | 0,8 | 0,6 | 320 |
| 4 | UC |  | - | X | - | 726 |  | 229 |  | 10 |  | 119 |  | 0,5 |  | 0 |
| 5 | none |  | - | X | - | 764 |  | 453 |  | 33 |  | 38 |  |  |  | 1280 |
| 6 | none |  | - | X | - | 656 |  | 379 |  | 63 |  | 69 |  | 0,9 |  | 0 |
| 7 | UC | psoriasis vulgaris | - | X | - | 451 | 97 | 162 | 24 | 16 | 17 | 61 | 22 | 1,0 | 0,1 | 320 |
| 8 | CD |  | - | X | - | 61 |  | 27 |  | 17 |  | 8 |  |  |  | 0 |
| 9 | UC |  | X | X | X | 160 | 70 | 174 | 16 | 36 | 23 | 52 | 20 | 0,4 | 0,5 | 160 |
| 10 | none |  | - | X | X | 500 | 112 | 80 | 13 | 100 | 23 | 150 | 30 | 3,0 | 1,5 | 0 |
| 11 | none |  | - | X | X | 1124 | 355 | 426 | 109 | 58 | 45 | 73 | 51 | 1,2 | 1,0 | 0 |
| 12 | UC | AIH | - | X | X | 750 | 120 | 622 | 49 | 76 | 31 | 81 | 35 | 0,8 | 1,1 | 320 |
| 13 | none | RA | X | X | X | 509 | 222 | 203 | 193 | 57 | 24 | 109 | 33 | 1,0 | 0,8 | 0 |
| 14 | CD |  | X | X | X | 190 | 169 | 245 | 175 | 106 | 39 | 84 | 51 | 0,8 | 0,7 | 0 |
| 15 | none | Churg-Strauss | X | X | X | 595 | 93 | 687 | 20 | 65 | 28 | 210 | 40 | 0,9 | 0,5 | 0 |
| 16 | UC |  | X | X | - | 361 | 91 | 600 | 19 | 37 | 19 | 134 | 21 | 0,7 | 0,9 | 80 |
| 17 | none |  | X | X | X | 160 | 151 | 57 | 15 | 26 | 19 | 33 | 13 | 0,6 | 0,6 | 0 |
| 18 | none |  | X | X | X | 556 | 120 | 1315 | 393 | 220 | 119 | 480 | 314 | 1,7 | 1,7 | 0 |
| 19 | CD |  | X | X | X | 907 | 733 | 297 | 166 | 109 | 66 | 214 | 131 | 3,3 | 1,4 | 160 |
| 20 | UC | ankylosing spondylitis | X | X | X | 88 | 63 | 186 | 25 | 39 | 21 | 74 | 27 | 0,4 | 0,1 | 160 |
| 21 | none |  | X | X | X | 62 | 51 | 89 | 6 | 65 | 27 | 167 | 32 | 0,8 | 1,1 | 640 |
| 22 | none |  | X | X | - | 162 | 139 | 232 | 46 | 52 | 41 | 68 | 46 | 0,9 | 0,2 | 40 |
| 23 | none |  | X | X | X | 191 | 124 | 216 | 68 | 43 | 16 | 66 | 20 | 1,0 | 0,6 | 0 |
| 24 | UC |  | X | X | X | 261 | 99 | 811 | 63 | 136 | 36 | 277 | 36 | 0,3 | 0,7 | 0 |
| 25 | none |  | X | X | - | 192 | 175 | 279 | 143 | 26 | 34 | 28 | 38 | 0,6 | 1,0 | 0 |
| 26 | none |  | X | X | - | 157 | 139 | 351 | 86 | 51 | 32 | 118 | 49 | 0,5 | 0,4 | 0 |
| 27 | UC |  | X | X | X | 299 | 90 | 97 | 67 | 51 | 28 | 40 | 66 | 4,1 | 0,2 | 40 |
| 28 | none |  | - | X | - | 240 |  | 180 |  | 49 |  | 38 |  | 3,2 |  | 0 |
| 29 | UC |  | X | X | - | 99 | 124 | 60 | 99 | 74 | 99 | 84 | 116 | 0,2 | 1,8 | 0 |
| 30 | UC |  | - | X | X | 202 | 89 | 20 | 10 | 28 | 14 | 34 | 13 | 0,5 | 0,7 | 320 |
| 31 | CD |  | X | - | X | 555 | 151 | 150 | 10 | 42 | 16 | 72 | 13 | 2,1 | 1,6 | 1280 |

| no | IBD | other AI  disease | PBMC  available | serum available | | AP (IU) ^a)^ | | γGT (IU) ^a)^ | | ASAT (IU) ^a)^ | | ALAT (IU) ^a)^ | | bilirubin (mg%) | | pANCA  rec titer |
| --- | --- | --- | --- | --- | --- | --- | --- | --- | --- | --- | --- | --- | --- | --- | --- | --- |
|  |  |  |  | before therapy | during therapy | before therapy | during therapy | before therapy | during therapy | before therapy | during therapy | before therapy | during therapy | before therapy | during therapy |  |
| 32 | UC | psoriasis vulgaris | X | - | X |  | 108 |  | 10 |  | 7 |  | 5 |  | 0,5 | 640 |
| 33 | none |  | X | - | X | 464 | 218 | 94 | 67 | 101 | 45 | 141 | 61 | 5,2 | 0,9 | 0 |
| 34 | UC |  | - | - | X | 1090 |  | 397 |  | 36 |  | 47 |  | 1,9 |  | 80 |
| 35 | UC |  | X | - | X | 232 | 155 | 206 | 22 | 114 | 34 | 78 | 44 | 3,1 | 2,4 | 40 |
| 36 | UC |  | - | - | X | 1135 |  | 58 |  | 45 |  | 72 |  | 8,6 |  | 80 |
| 37 | none |  | X | - | X | 325 | 117 | 341 | 148 | 123 | 32 | 176 | 47 | 1,0 | 0,6 | 0 |
| 38 | UC |  | - | - | X | 823 |  | 353 |  | 34 |  | 32 |  | 1,0 |  | 0 |
| 39 | none |  | - | - | X |  | 239 |  | 124 |  | 21 |  | 35 |  | 0,8 | 0 |
| 40 | UC |  | X | - | X | 126 | 71 | 96 | 88 | 17 | 35 | 22 | 38 | 1,4 | 1,0 | 40 |
| 41 | CD |  | X | - | X |  | 242 |  | 87 |  | 10 |  | 15 |  | 1,0 | 0 |
| 42 | none |  | X | - | X | 375 | 94 | 598 | 78 | 137 | 45 | 119 | 33 | 1,8 | 3,5 | 0 |
| 43 | UC | AIH | X | - | X | 306 | 195 | 100 | 63 | 22 | 69 | 20 | 53 | 0,4 | 1,2 | 0 |
| 44 | none |  | X | - | X |  | 159 |  | 394 |  | 24 |  | 136 |  | 0,6 | 0 |
| 45 | UC |  | - | - | X | 747 |  | 87 |  | 65 |  | 69 |  | 2,4 |  | 320 |
| 46 | none |  | - | - | X | 862 |  | 641 |  | 89 |  | 200 |  | 7,3 |  | 0 |
| 47 | CD |  | - | - | X |  | 146 |  | 52 |  | 99 |  | 252 |  | 1,2 | 0 |
| 48 | UC |  | X | - | X |  | 269 |  | 43 |  | 23 |  | 22 |  | 0,8 | 0 |
| 49 | none |  | X | - | X | 716 | 227 | 331 | 174 | 62 | 155 | 130 | 122 | 1,8 | 1,5 | 0 |
| 50 | none |  | X | - | X | 306 | 112 | 59 | 18 |  | 37 |  | 39 | 2,2 | 1,3 | 640 |
| 51 | none |  | - | - | X | 1092 | 155 | 102 | 49 | 43 | 38 | 53 | 65 | 1,6 | 0,8 | 0 |
| 52 | CD |  | X | - | X |  | 250 |  | 136 |  | 29 |  | 32 |  | 0,8 | 0 |
| 53 | UC |  | X | - | X |  | 273 |  | 105 |  | 34 |  | 59 |  | 0,8 | 640 |

CU – ulcerative colitis; CD – Crohn’s disease; AI – autoimmune; PBMC – peripheral blood mononuclear cells; AP – alkaline phosphatase; gGT gamma-glutamyltransferase; ASAT – aspartate amino transferase; ALAT – alanine amino transferase

^a)^ In patients during UDCA therapy levels of AP, γGT, ASAT and ALAT had significantly decreased as compared to before therapy (p < 0.001). Bilirubin levels were not significantly influenced.
