# Supplementary material for: Sulphite oxidase (SO) – a mitochondrial autoantigen as target for humoral and cellular immune reactions in primary sclerosing cholangitis
Source: BMC Gastroenterol. 2018 May 2;18:58. doi: 10.1186/s12876-018-0787-x (PMC5932765; doi:10.1186/s12876-018-0787-x)
Supplement: Supplementary file 3 — Overlapping synthetic peptides of human SO protein chain. (PDF 193 kb) [file 12876_2018_787_MOESM3_ESM.pdf]

Additional file 3 Overlapping synthetic peptides of human SO protein chain

	Peptide	Position (aa)	Sequence
	1	1-25	MGTLLGLGAVLAYQDHR**CRAAQEST**
	2	18-43	**CRAAQEST**HIYTKEEVS**SHTSPETG**
	3	35-59	**SHTSPETG**IWVTLGSEV**FDVTEFVD**
SO I	4	52-76	**FDVTEFVD**LH*PGGPSKL**MLAAGGPL**
	5	69-93	**MLAAGGPL**EPFWALYAV**H** * **NQSHVRE**
	6	86-110	**H** * **NQSHVRE**LLAQYKIGE**LNPEDKVA**
	7	103-127	**LNPEDKVA**PTVETSDPY**ADDPVRHP**
	8	120-144	**ADDPVRHP**ALKVNSQRP**FNAEPPPE**
	9	137-161	**FNAEPPPE**LLTENYITP**NPIFFTRN**
	10	154-178	**NPIFFTRN**HLPVPNLDP**DTYRLHVV**
	11	171-195	**DTYRLHVV**GAPGGQSLS**LSLDDLHN**
	12	188-212	**LSLDDLHN**FPRYEITVT**LQC** ** **AGNRR**
SO II	13	205-229	**LQC** ** **AGNRR**SEMTQVKEV**KGLEWRTG**
	14	222-246	**KGLEWRTG**AISTARWAG**ARLCDVLA**
	15	239-263	**ARLCDVLA**QAGHQLCET**EAHVC** ** **FEG**
	16	256-280	**EAHVC** ** **FEG**LDSDPTGTA**YGASIPLA**
	17	273-297	**YGASIPLA**RAMDPEAEV**LLAYEMNG**
	18	290-314	**LLAYEMNG**QPLPRDHGF**PVRVVVPG**
	19	307-331	**PVRVVVPG**VVGARHVKW**LGRVSVQP**
	20	324-348	**LGRVSVQP**EESYSHWQR**RDYKGFSP**
	21	341-365	**RDYKGFSP**SVDWETVDF**DSAPSIQE**
	22	358-382	**DSAPSIQE**LPVQSAITE**PRDGETVE**
SO III	23	375-399	**PRDGETVE**SGEVTIKGY**AWSGGGRA**
	24	392-416	**AWSGGGRA**VIRVDVSLD**GGLTWQVA**
	25	409-433	**GGLTWQVA**KLDGEEQRP**RKAWAWRL**
	26	426-450	**RKAWAWRL**WQLKAPVPA**GQKELNIV**
	27	443-467	**GQKELNIV**CKAVDDGYN**VQPDTVAP**
	28	460-484	**VQPDTVAPIWNLRGVLSNAWHRVHV**
	29	464-488	**TVAPIWNLRGVLSNAWHRVHV**YVSP

The overlapping amino acids are marked by bold letters.

*: binding sites for iron as heme axial ligand in position aa 61 and aa 86.

**: binding sites for molybdenum pterin in position aa 207 and aa 260.
