# Supplementary material for: Sulphite oxidase (SO) – a mitochondrial autoantigen as target for humoral and cellular immune reactions in primary sclerosing cholangitis
Source: BMC Gastroenterol. 2018 May 2;18:58. doi: 10.1186/s12876-018-0787-x (PMC5932765; doi:10.1186/s12876-018-0787-x)
Supplement: Supplementary file 4 — Receiver operating curve (ROC) analysis for IgG-antibodies to the four SO-proteins comparing sera from PSC patients with those from healthy donors, PBC, AIH, alcoholic liver disease (ALD), viral hepatitis, ulcerative colitis (CU), Crohn disease (CD), and collagen disorders. (PDF 257 kb) [file 12876_2018_787_MOESM4_ESM.pdf]

Additional file 4

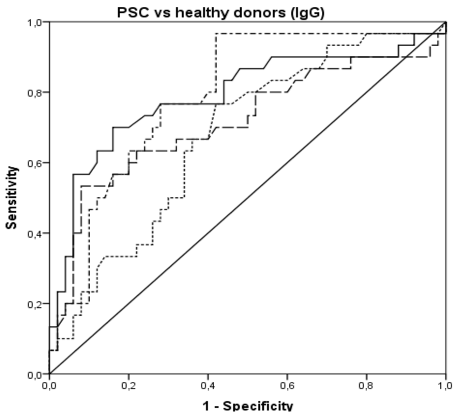

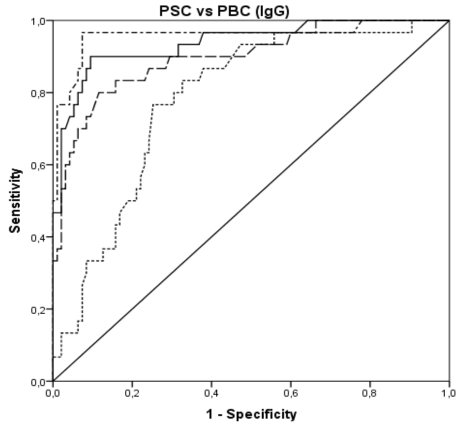

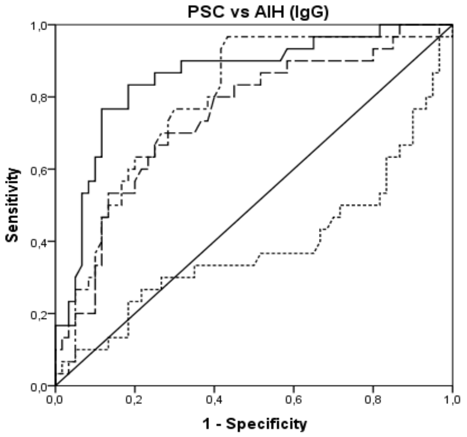

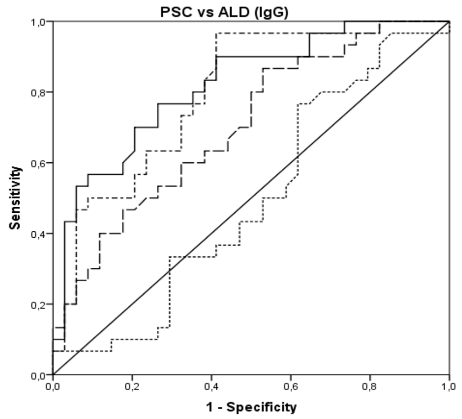

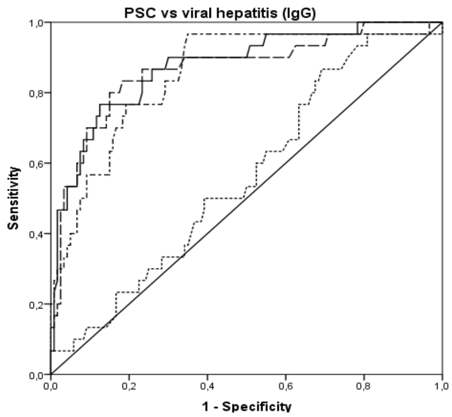

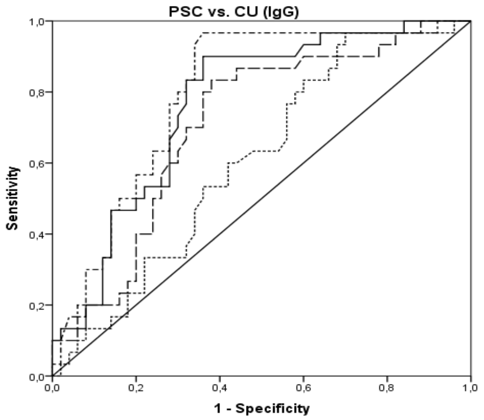

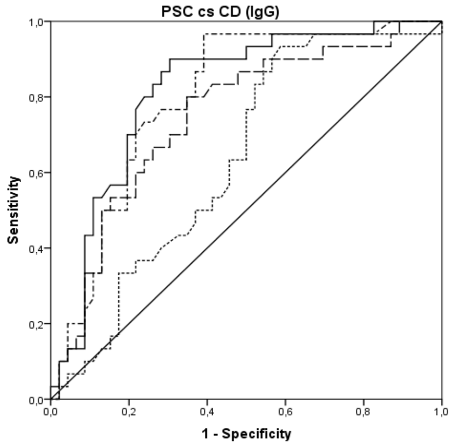

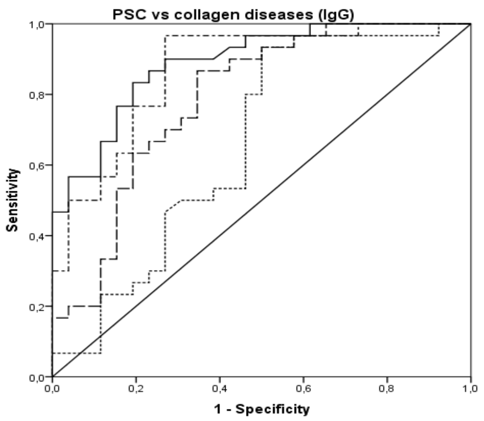

Receiver operating curve (ROC) analysis for IgG-antibodies to the four SO-proteins comparing sera from PSC patients with those from healthy donors, PBC, AIH, alcoholic liver disease (ALD), viral hepatitis, ulcerative colitis (CU), Crohn disease (CD), and collagen disorders (B) ―― SO-fl; — · SO-I – – SO-II ······ SO-III

Significances for anti-SO antibodies of the **IgG-type** comparing PSC-patients and patients with other disorders by ROC curves calculating area under the curves (AUC)

PSC vs.	SO-fl		SO-I		SO-II		SO-III	
	AUC	p	AUC	p	AUC	p	AUC	p
Healthy donors	0,79	**0,000**	0,79	**0,000**	0,71	**0,001**	0,68	**0,009**
PBC	0,94	**0,000**	0,96	**0,000**	0,90	**0,000**	0,78	**0,000**
AIH	0,86	**0,000**	0,79	**0,000**	0,75	**0,000**	0,39	0,077
ALD	0,82	**0,000**	0,80	**0,000**	0,70	**0,005**	0,49	0,936
Viral hepatitis	0,88	**0,000**	0,85	**0,000**	0,87	**0,000**	0,56	0,302
Ulcerative colitis (CU)	0,76	**0,000**	0,80	**0,000**	0,71	**0,002**	0,61	0,111
Crohn disease (CD)	0,816	**0,000**	0,80	**0,000**	0,75	**0,000**	0,63	**0,049**
Collagen disorders	0,89	**0,000**	0,87	**0,000**	0,79	**0,000**	0,66	**0,041**
PSC treated	0,70	**0,012**	0,69	**0,021**	0,62	0,141	0,61	0,181

Significances for anti-SO antibodies of the **IgM-type** comparing PSC-patients and patients with other disorders by ROC curves (not shown as a figure) calculating area under the curves (AUC)

PSC vs.	SO fl		SO I		SO II		SO III	
	AUC	p	AUC	p	AUC	p	AUC	p
Healthy donors	0,64	**0,045**	0,55	0,484	0,67	**0,011**	0,75	**0,000**
PBC	0,95	**0,000**	0,87	**0,000**	0,97	**0,000**	0,97	**0,000**
AIH	0,82	**0,000**	0,76	**0,000**	0,83	**0,000**	0,90	**0,000**
ALD	0,79	**0,000**	0,71	**0,004**	0,80	**0,000**	0,85	**0,000**
Viral hepatitis	0,81	**0,000**	0,71	**0,000**	0,80	**0,000**	0,83	**0,000**
Ulcerative colitis	0,75	**0,000**	0,66	**0,015**	0,80	**0,000**	0,813	**0,000**
Crohn disease	0,72	**0,001**	0,65	**0,034**	0,68	**0,009**	0,801	**0,000**
Collagen disorders	0,95	**0,000**	0,85	**0,000**	0,93	**0,000**	0,95	**0,041**
PSC treated	0,75	**0,002**	0,72	**0,007**	0,75	**0,002**	0,75	**0,002**

AUC = area under the curve
