# Supplementary material for: Sulphite oxidase (SO) – a mitochondrial autoantigen as target for humoral and cellular immune reactions in primary sclerosing cholangitis
Source: BMC Gastroenterol. 2018 May 2;18:58. doi: 10.1186/s12876-018-0787-x (PMC5932765; doi:10.1186/s12876-018-0787-x)
Supplement: Supplementary file 5 — Activity of IgG-antibodies against the four SO-proteins in sera from untreated PSC-patients without and with IBD as well as in patients with pure IBD as determined by ELISA. Individual values (•) and median (―) are given. p < 0.05 (with post-hoc tests Tukey and Bonferroni). (PDF 164 kb) [file 12876_2018_787_MOESM5_ESM.pdf]

Additional file 5

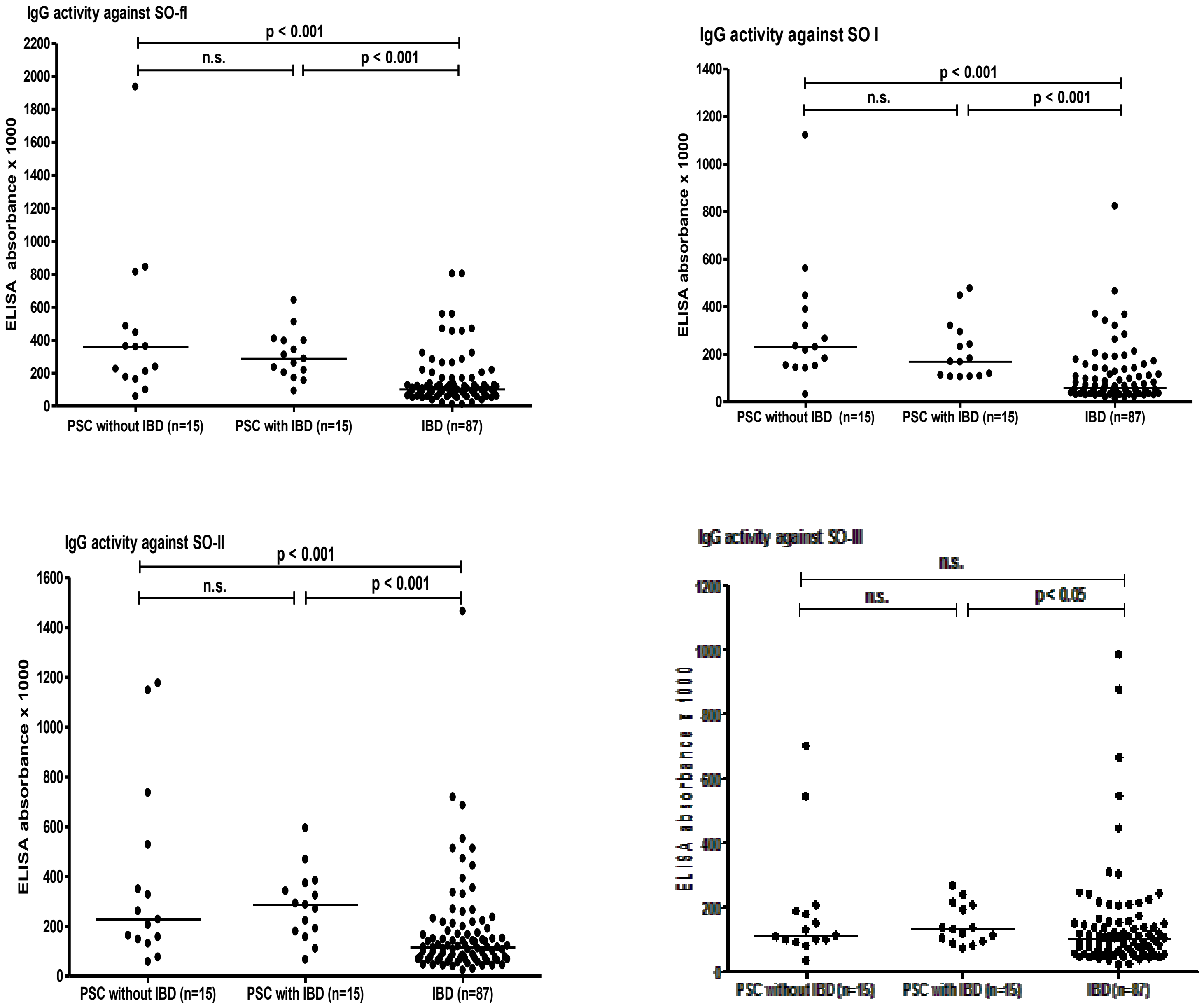

Activity of IgG-antibodies against the four SO-proteins in sera from untreated PSC-patients without and with IBD as well as in patients with pure IBD as determined by ELISA. Individual values (•) and median (—) are given. $p < 0.05$ (with post-hoc tests Tukey and Bonferroni).
